# Multisensory correlations—Not tactile expectations—Determine the sense of body ownership

**DOI:** 10.1371/journal.pone.0213265

**Published:** 2019-02-28

**Authors:** Arvid Guterstam, Dennis E. O. Larsson, Hugo Zeberg, H. Henrik Ehrsson

**Affiliations:** 1 Department of Neuroscience, Karolinska Institutet, Stockholm, Sweden; 2 Princeton Neuroscience Institute, Princeton University, Princeton, New Jersey, United States of America; Birkbeck University of London, UNITED KINGDOM

## Abstract

Can the mere expectation of a sensory event being about to occur on an artificial limb be sufficient to elicit an illusory sense of ownership over said limb? This issue is currently under debate and studies using two different paradigms have presented conflicting results. Here, we employed the two relevant paradigms, namely, the magnetic touch illusion and the “tactile expectation” version of the rubber hand illusion, to clarify the role of tactile expectations in the process of attributing ownership to limbs. The illusory senses of ownership and ‘magnetic touch’ were quantified using questionnaires, threat-evoked skin conductance responses and a combination of motion tracking synchronized with real-time subjective ratings and skin conductance. The results showed that the magnetic touch illusion was dependent on concurrent visual and tactile stimulation and that visually induced tactile expectations alone were insufficient. Moreover, in this study, tactile expectations were not associated with the rubber hand illusion, neither in terms of subjective ratings nor skin conductance changes. Together, these findings contradict the notion that the brain uses predictions of upcoming sensory events to determine whether or not a limb belongs to the self, and, instead, emphasize the importance of correlated multisensory information.

## Introduction

When reaching out your hand to catch a ball thrown at you, the brain forms predictions about the upcoming tactile event based on the visual input from the incoming ball and your own hand. In certain situations, sensory predictions may even attenuate the perception of the subsequent tactile event. For instance, it has been shown that a self-generated tactile stimulus is perceived as weaker [1,2] and less ticklish [3] than the same stimulus generated externally. In the body ownership literature, there is currently an ongoing debate relating to whether sensory predictions may alter the perception of an artificial limb. Specifically, it has been argued that the mere expectation of a tactile event being about to occur on a rubber hand is sufficient to elicit a sense of ownership over that limb [4–6], which contradicts the leading view in the field that ownership illusions require correlated sensory signals from at least two sensory modalities (or somatosensory submodalities) [7–11]. However, a recent study from our group investigating the “magnetic touch illusion” failed to find an effect of such “tactile expectations” on limb ownership [12], sparking a scientific debate [13]. In this study, we set out to test whether tactile expectations play any role in the generation of magnetic touch illusion [12,14] as well as replicating the basic tactile expectation effect on ownership [4–6].

In the original rubber hand illusion, temporally and spatially congruent visual information from a rubber hand being touched and tactile information from a participant’s real hand, which is occluded from view, lead to the illusion of ownership of the rubber hand [15]. This illusion elegantly demonstrated that the integration of correlated signals from different sensory modalities is a key mechanism for body ownership. This notion was recently challenged by a study claiming that it is possible to elicit ownership of the rubber hand by merely observing a visual stimulus approaching—without touching—the rubber hand [4]. In that study, the visual stimulus consisted of the experimenter’s hand slowly (2cm/s) approaching the rubber hand as if about to touch it, starting from a distance of 70 cm and stopping 15 cm above it, while the participant’s real hand was hidden under a small table immediately below the rubber hand. Importantly, ownership was only elicited in the condition when the rubber hand was placed in an anatomically plausible position, but not when it was rotated by 180°, replaced by a block of wood or when just viewing a rubber hand without any approaching visual stimulus [4]. Furthermore, in the tactile expectation condition, the approach of the visual stimulus was coupled with an increase in skin conductance for distances ≤30 cm. The skin conductance change magnitude correlated with the subjectively reported ownership, and was taken as an objective measure of successful induction of rubber hand ownership [4].

In a recently published study, we characterized a novel version of the rubber hand illusion termed the “magnetic touch illusion” [12]. In this illusion, a visible gap between the touching object (a brush) and the rubber hand is introduced, which, in addition to the illusion of ownership of the rubber hand, results in the illusory sensation of there being a ‘magnetic force’ or ‘force field’ between the brush and the rubber hand [12]. In two behavioral experiments of that study (Experiments 2a and 2b), we used questionnaires and an inter-manual pointing task that quantifies the so-called proprioceptive drift [12], to investigate whether tactile expectations contributed to the sense of magnetic touch. Specifically, three conditions were included: (i) visual stimulation in the form of a brush slowly approaching the rubber hand in a continuous ‘tapping’ motion in combination with synchronous tapping on the real hand (*visuo-tactile*); (ii) only visual stimulation featuring the same approaching ‘tapping’ motion toward the rubber hand (*visual only—hand approaching*); and (iii) only visual stimulation featuring a brush held at a fixed position (*visual only—hand static*). The results showed that only the *visuo-tactile* condition was associated with rubber hand ownership and illusory magnetic touch, while there was no illusion and no significant differences between *visual only—approaching brush* and *visual only—static brush* conditions. These findings thus contradicted the notion the tactile expectations alone (i.e., the *visual only—approaching brush* condition) can elicit ownership. However, as pointed out in a commentary [13], there was one, perhaps crucial, difference between our experimental setup and the one used in [4]: in [4], the rubber hand was displaced vertically relative to the real hand, while we displaced it horizontally [12]. Ferri and Constantini (2016) suggested that the horizontal displacement prevents the formation of tactile expectations with regard to the real hand, because the approaching object moves along a path toward the rubber hand that will never, even potentially, touch the real hand. They proposed that tactile expectations need to be formed for both the real and rubber hand simultaneously for the rubber hand illusion–and the magnetic touch illusion–to be induced, i.e., by using a path of the approaching object that is compatible with a collision with both the real and the rubber hands [13].

In the present study, we used a combination of motion tracking synchronized with the recording of skin conductance and real-time subjective ratings to test this alternative explanation. Crucially, we used a vertical, instead of horizontal, displacement of the real and rubber hands throughout. In addition, we aimed at replicating the basic tactile expectation effect by mimicking the experimental setup of [4]. To this end, we included four experimental conditions: (1) visual stimulation in the form of a brush slowly approaching the rubber hand in a continuous, ‘tapping’ motion in combination with synchronous tapping on the real hand (*visuo-tactile*); (2) only visual stimulation featuring the same approaching ‘tapping’ motion toward the rubber hand (*visual only—brush approaching*); (3) only visual stimulation featuring the experimenter’s hand slowly approaching the rubber hand in a continuous movement (*visual only—hand approaching*); or (4) only visual stimulation consisting of the experimenter’s hand held at a fixed position 30 cm above the rubber hand. If tactile expectations alone indeed are sufficient to induce ownership sensations, then not only the *visuo-tactile* but also the *visual only—brush approaching* and *visual only—hand approaching* conditions should be associated with the magnetic touch illusion, because these two conditions should induce tactile expectations for both the real and rubber hands. However, if correlated sensory stimulation from two sensory modalities is obligatory for ownership, then only the *visuo-tactile* condition should be associated with the illusion.

## Methods

### Participant information

We recruited a total of 24 healthy adult volunteers (mean age±SEM = 25.4±0.7 years), of which 10 were female, and 23 were right-handed. All subjects gave written informed consent prior to participation, and the Regional Ethical Review Board of Stockholm approved all of the experimental procedures. The experiment was performed in accordance with relevant guidelines and regulations.

### Experimental setup and illusion induction procedure

**Fig 1:**
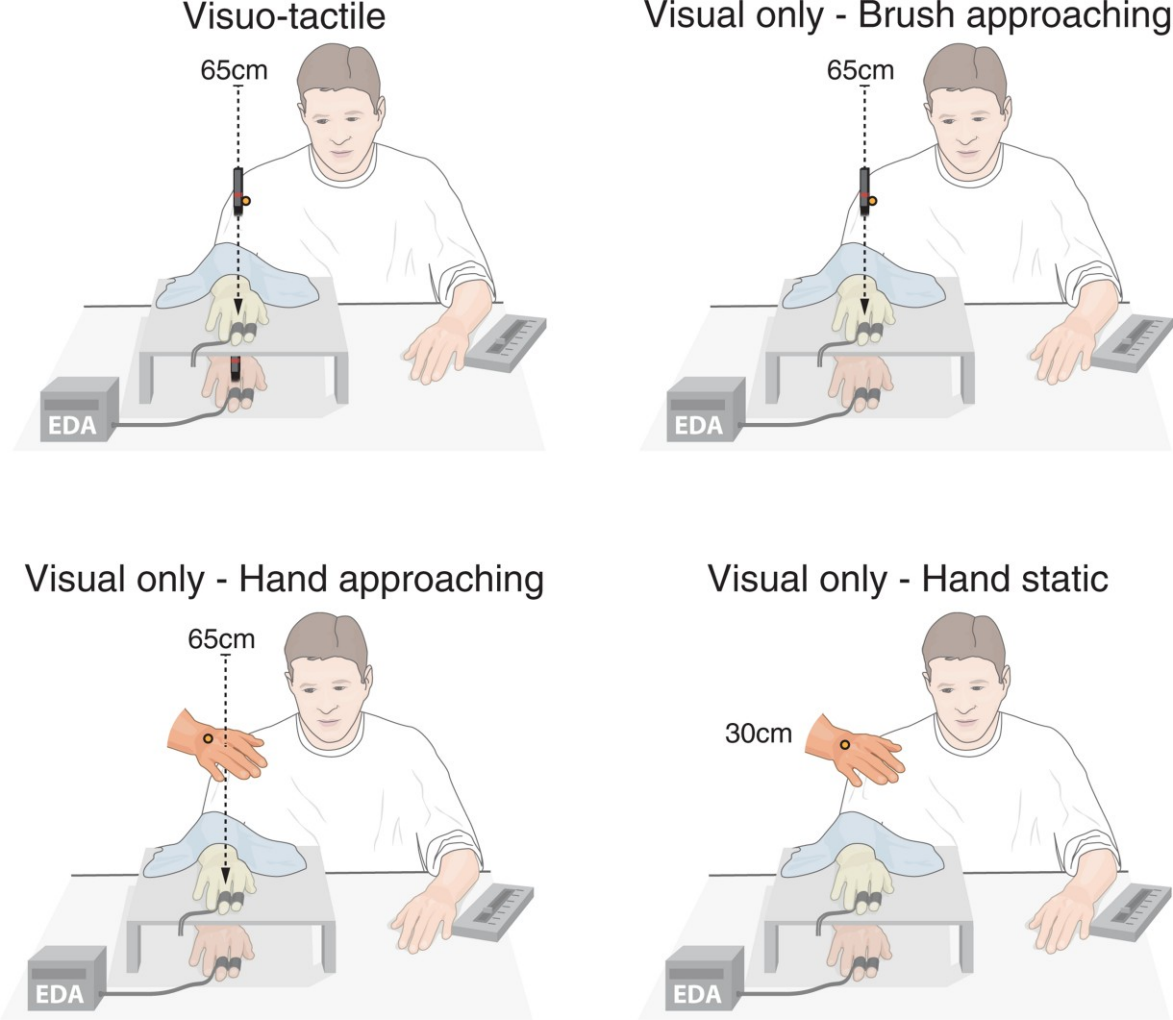
Experimental setup. To examine the role of tactile expectations on the magnetic touch illusion and on limb ownership in general, four experimental conditions were included: Visual stimulation in the form of a brush slowly approaching the rubber hand in a continuous ‘tapping’ motion, while synchronous taps were delivered to the hidden real hand using an identical brush (*visuo-tactile*). This condition should elicit the magnetic touch illusion [12]. The *visual only—brush approaching* condition featured identical visual stimulation as in *visuo-tactile*, but no tactile stimulation of the real hand. The *visual only—hand approaching* condition consisted of the experimenter’s hand slowly approach the rubber hand, as in [4]. Theoretically, both of these conditions should induce tactile expectations, because the trajectory of the visual stimulus is along a path on “collision course” with both the real and rubber hands [13]. In the *visual only—hand static* condition, the experimenter’s hand was held in a fixed position 30cm above the rubber hand. This condition should not be associated with tactile expectations and served as a baseline condition to control for the effect of simply looking at a rubber hand. *EDA = electrodermal activity. Illustration credit: Mattias Karlén.

The experiments took place in a soundproof testing room (40-decibel noise reduction). The participants sat on a comfortable chair and rested their arms on a table in front of them, and the experimenter sat opposite them. The participants’ right arm was placed below a small table and was thus hidden from view. A cosmetic right male rubber hand (Fillauer Europe AB, Sollentuna, Sweden) was placed on top of the small table, 15 cm above the real right hand (Fig 1). The magnetic touch illusion was elicited in the visuo-tactile condition by applying focal touches to the participant’s hidden right hand while simultaneously moving another brush, fully visible to the participant, and making small tapping movements while approaching the rubber hand. The motion started at 65 cm and stopped a couple of cm above the rubber hand. A small sensor (see section ‘Motion tracking’ below for details) that continuously recorded three-dimensional spatial coordinates was attached to the tip of brush moving in mid-air. The touches were applied to the dorsal surface of the hand and the proximal phalanges of digits I-IV of the participant’s right hand and corresponding locations above the rubber hand. Approximately 60 ‘taps’ were applied per minute in the visuo-tactile and visual only conditions.

### Experimental conditions

To test our hypotheses, we included four experimental conditions. In the *visuo-tactile* condition, which was designed to elicit the magnetic touch illusion [12], the participants’ hidden real hand was touched in synchrony with visual stimulation in the form of the experimenter’s hand holding a brush and making small tapping movements while approaching the rubber hand at the velocity of approximately 2 cm/s (Fig 1). The *visual only—brush approaching* featured the same visual stimulation as the *visuo-tactile* condition, but without the tactile stimulation of the real hand (Fig 1). In theory, this condition should induce tactile expectations, given that the hand holding the brush is continuously approaching the rubber hand as well as the vertically displaced hidden real hand [4–6,13]. In order to imitate the original tactile expectation paradigm as closely as possible [4], we also included a *visual only—hand approaching* condition in which the experimenter’s hand made a continuous, slow movement (2 cm/s) toward the rubber hand without holding a brush (Fig 1). Finally, to isolate the effect of the approaching movement of the hand, which should be crucial for eliciting tactile expectations, we included a forth control condition in which the experimenter held his hand in a static position approximately 30 cm above to rubber hand *visual only—hand static*; Fig 1). Notably, the *visual only—hand approaching* and *visual only—hand static* conditions featured only visual stimulation; no touches were applied to the real hand.

### Experimental design

The experiment consisted of three main parts. In the first part of the experiment, the participants were exposed to the classical rubber hand illusion [15] and the original magnetic touch illusion [12] in two separate 2-min blocks (in a counter-balanced order across participants). In the magnetic touch illusion condition, brushstrokes were applied in mid-air approximately 5 cm above the surface of the rubber hand, while all other experimental factors were identical to the classical rubber hand illusion condition. Questionnaires that assessed the illusory sense of ownership and magnetic touch (adopted from the [12,15]) were administered immediately after each block. The purpose of including these two initial experimental blocks was two-fold. First, we reasoned that the experience of the classical versions of the illusions would give the participants an internal reference as to how illusory limb ownership and magnetic touch feels like, which would be helpful for the subsequent experimental blocks where they were instructed to continuously rate the feeling of magnetic touch in real-time. Second, it allowed us to calculate the proportion of responders to the classical rubber hand and magnetic touch illusions. Indeed, 23 participants (96%) experienced the rubber hand illusion (mean questionnaire rating of the key ownership statement S5 ± SD: 2.3 ± 0.8) and 19 participants (79%) experienced the magnetic touch illusion (mean questionnaire rating of the key magnetic touch statement S1 ± SD: 1.2 ± 0.8), using the definition of illusion responder as someone who rates statement S1 and S5, respectively, at least +1 (see section “Questionnaire” below for further details). Furthermore, female participants in the classical rubber hand illusion experienced ownership of the male rubber hand as strongly as the male participants (mean rating of S5: 2.3 versus 2.2; t_22_ = -0.26, p = 0.801, *d* = -0.11, two-samples t-test). All 24 participants, including the one who did not experience the classical rubber hand illusion, were included in the second part of the experiment, which is described below. See S1 Fig for the complete questionnaire results for this part of the experiment.

In the second part of the experiment, the participants were exposed to the *visuo-tactile*, *visual only -brush approaching*, *visual only—hand approaching* and *visual only—hand static* conditions within four separate 2-min blocks in a counterbalanced order. Each block consisted of four consecutive approaching movements, each of which took approximately 30 s, where the visual stimulus started at 65 cm and stopped at 2–3 cm above the rubber hand (mean velocity approximately 2 cm/s). In the *hand static* condition, the experimenter’s hand was held in a fixed position 30 cm above the rubber hand for 30 s, in four consecutive periods. Immediately after each of the four approaching movements/static periods, the experimenter subjected the rubber hand to a physical threat in the form of a small knife that made a ‘cutting’ motion above the dorsum of the rubber hand. As shown by the motion tracking data of the threat event (S2 Fig), the experimenter performed the knife threatening motion very consistently across trials, conditions and participants. The presentation of the knife was flagged in the data file by the experimenter pressing a foot pedal, and the duration of the threat stimulus was approximately 2 s. In total, there were four threat events per condition. After each 2-min block, the participants were asked to fill out a questionnaire consisting of eight statements regarding the subjective feeling of ownership and magnetic touch (see *Questionnaires* below for details).

In the third and final part of the experiment, the *visuo-tactile*, *visual only—brush approaching*, *visual only—hand approaching* and *visual only -hand static* conditions were again presented in a counterbalanced order within four separate 2-min-blocks, each comprising four approaching movements/static periods. This time, the participants were instructed to continuously rate their subjective feeling of magnetic touch using a sliding bar (TSD115 Variable assessment transducer, BIOPAC, Goleta, California, USA) placed in their left hand. Specifically, they were instructed to rate the statement *“It seemed as though there was a ‘magnetic force’ or ‘force field’ between the brush and the rubber hand”* on a scale between -3 (*“I disagree completely”*) and +3 *(“I agree completely”*). No knife threat was presented during this part of the experiment. However, we recorded the continuous level of skin conductance, in line with the use of skin conductance measurements in [4]).

### Questionnaires

The participants were asked to affirm or deny different statements reflecting potential perceptual effects using a seven-point visual analogue scale that ranged from -3 to +3. The participants were informed that -3 indicated “I completely disagree”, +3, indicated “I agree completely”, and 0 indicated “I do not know, I can neither agree nor disagree”. Statements 1 and 2 (S1-S2) were designed to examine the sensation of magnetic touch, whereas statements 3 and 4 (S3-S4) served as controls for suggestibility and task compliance. Statements 5 and 6 (S5-S6) were designed to examine the feeling of rubber hand ownership; statements 7 and 8 (S7-S8) serving as controls. See Table 1 for the statements.

**Table 1:** Questionnaire statements.

**Magnetic touch sensation—illusion statements**
S1. It seemed as though there was a "magnetic force" or "force field" between the brush /experimenter's hand and the rubber hand.
S2. It felt as if the brush / experimenter's hand I saw caused the sensation of touch on the rubber hand below.
**Magnetic touch sensation—control statements**
S3. It seemed as though there was a "magnetic force" or "force field" directly connecting the rubber hand to my real hand.
S4. It seemed as if the rubber hand was drifting towards my real hand.
**Rubber hand ownership—illusion statements**
S5. It felt as if the rubber hand were my hand.
S6. I felt touch on the rubber hand.
**Rubber hand ownership—control statements**
S7. It felt as if I had two right hands.
S8. I felt a painful touch.

### Motion tracking

We used motion tracking based on electromagnetic technology (Polhemus FASTRAK, Vermont, USA). A small sensor that continuously recorded its three-dimensional spatial coordinates at the rate of 120 Hz was attached to either the tip of the brush moving in mid-air (in the *visuo-tactile* and *visual only—brush approaching* conditions) or on the experimenter’s hand (in the *hand approaching* and *hand static* conditions). The experimenter controlled the recording of motion tracking data by pressing a foot pedal. A data file containing the X, Y, and Z coordinates for the brush sensor was created for each individual repetition of every condition, for all of the participants.

### Skin conductance measurements

The skin conductance was recorded with a Biopac System MP150 and following standard published guidelines [16]. The two recording electrodes (Biopac TSD203) were attached to the middle phalanges of the index and middle fingers of the participants’ right hand and recorded the skin conductance at a frequency of 200 Hz. To match the visual appearance of the rubber hand with the sensory input from the hidden real hand, two identical electrodes were attached to the corresponding fingers of the rubber hand.

**Fig 2:**
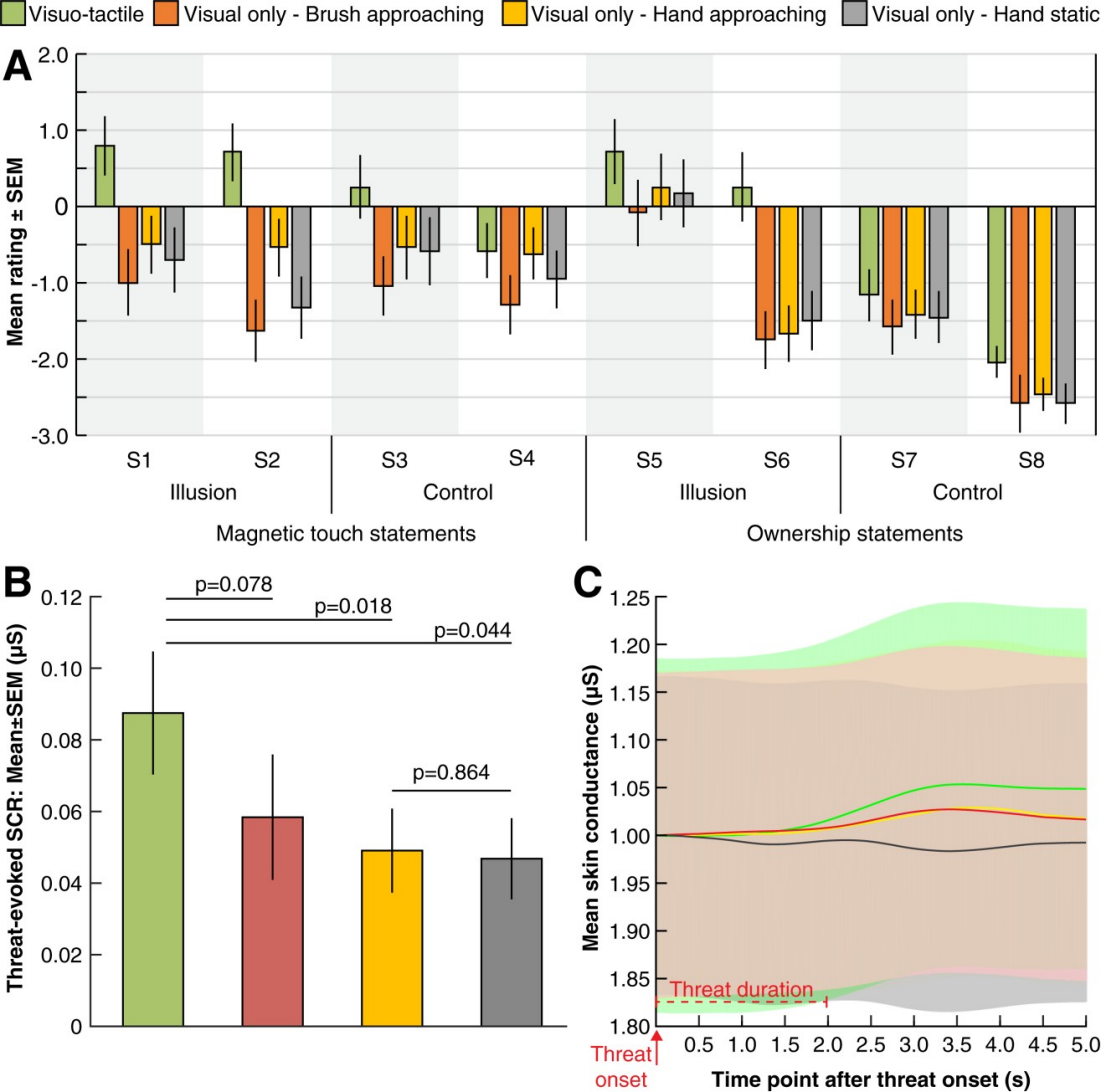
(**A**) **Questionnaire results.** Ratings of the questionnaire statements (Table 1) for each of the four experimental conditions. The error bars denote the SEM. (**B**) Mean threat-evoked skin conductance response (SCR), which was defined as the difference in conductance between the first moment the participant observed the knife threat and the peak in conductance occurring within 5 s. The error bars denote the SEM. (**C**) The temporal profiles of the threat-evoked SCRs, normalized relative to the conductance at t = 0s. The duration of the threat event was 2 s.

**Fig 3:**
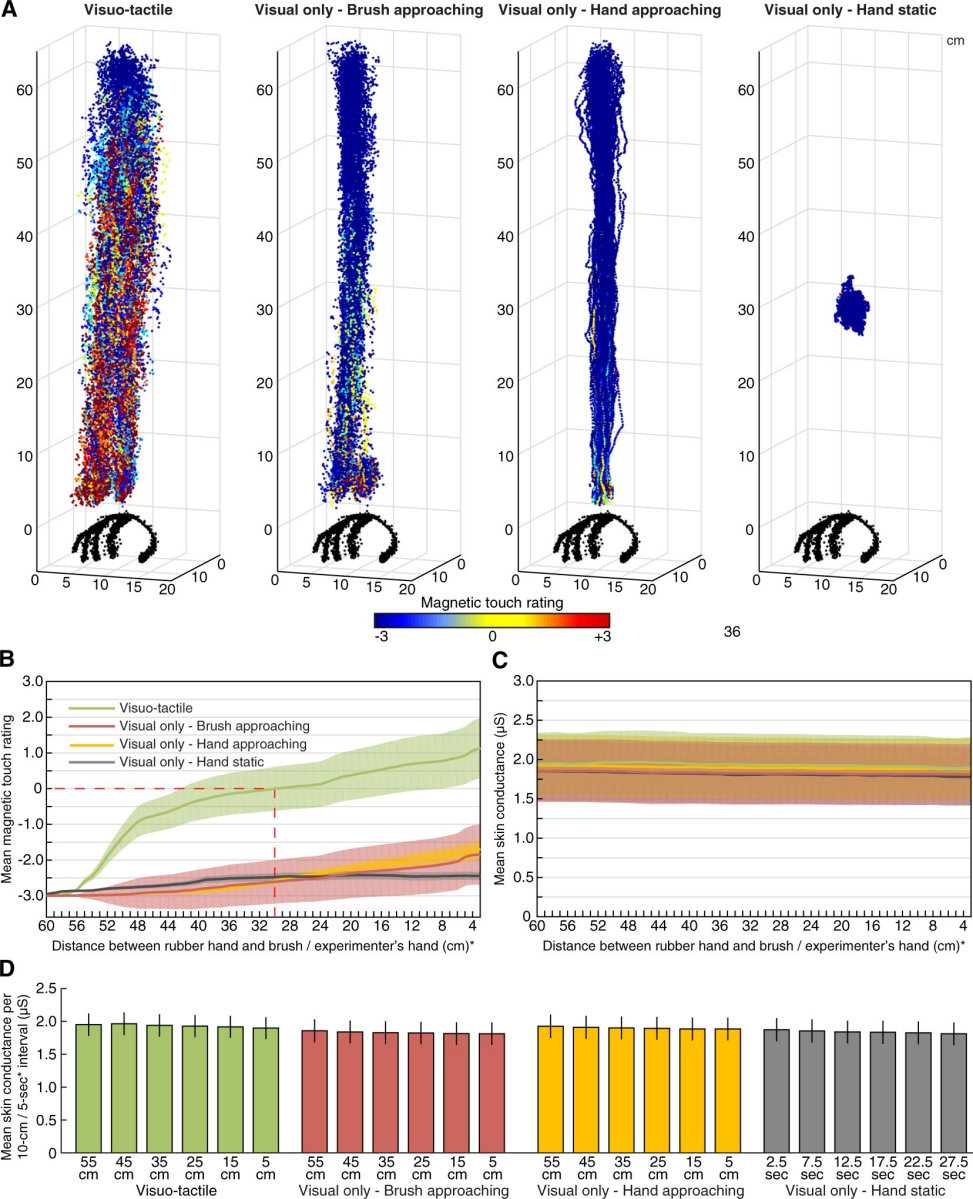
(**A**) **Motion tracking results**. All motion tracking data points (x-, y- and z-coordinates), color-coded according to the real-time rating of illusory magnetic touch, are shown for all participants and each condition. The surface of the rubber hand was also mapped and is shown in black. (**B**) **Real-time illusion vividness ratings**. The strength of the magnetic touch illusion, averaged for each 1-cm-interval between 60 and 3cm, is shown for each condition separately. In conjunction with visual inspection of the graphs in panel A, these results show that the illusion was present in the *visuo-tactile* condition, but in neither of the conditions involving tactile expectations (*brush* and *hand approaching*), nor in the baseline condition consisting of simply looking at a rubber hand (*hand static*). (**C**) **Skin conductance as a function of distance**. None of the conditions were associated with a change in skin conductance level as a function of the distance between the rubber hand and the approaching object. (**D**) Average skin conductance for every 10-cm-interval. Error bars denote the SEM. *In the *visual only—hand static* condition, during which no approaching movement was performed, the ratings (panel B) and skin conductance (panel C) are plotted against time (average over each 0.5-s interval between 0-29s) instead of distance.

We measured both the threat-evoked SCR (Fig 2B and 2C) and continuous skin conductance (Fig 3) for part two and three of the experiment (see *Experimental design* above). The threat-evoked SCR is an established proxy of illusory body ownership [17,18], while a continuous increase in skin conductance with decreasing distance between the approaching object and the rubber hand was found to be specific to the tactile expectation effect in a previous study [4]. In our analyses, the raw data was kept at the recording frequency (200 Hz), and no high- or low-pass filters were applied. The threat-evoked SCR was defined as difference in conductance between the onset time of the threat (i.e., the first moment that the knife entered the participant’s visual field, which was indicated by a foot pedal press) and the peak of the conductance that occurred within 5 seconds. The average of all responses for each participant, including those in which no increase in amplitude was apparent, was separately calculated for each condition, and this value was taken as the SCR magnitude [16]. Thereafter, the SCR magnitudes for all the participants were compared statistically across different conditions as described in the Results section.

For the continuously recorded skin conductance data, we first averaged the data for every 1-cm-interval between 60 and 3 cm (in the hand static condition, we averaged every 0.5-s-interval between 0–29 s, instead), for display purposes (Fig 3C). In our statistical analysis, we averaged the level of skin conductance for every 10-cm-interval between the distances 60 and 3 cm (“55 cm” = average across 51–60 cm; “45 cm” = average across 41–50 cm; “35 cm” = average across 31–40 cm; “25 cm” = average across 21–30 cm; “15 cm” = average across 11–20 cm; “5 cm” = average across 3–10 cm; see Fig 3D). Because the velocity of the approaching movement was approximately 2 cm/s, each 10-cm interval represented a time duration of about 5 s. In the *hand static* condition, we averaged the skin conductance for every 5-s interval between 0 and 30 s.

### Data synchronization

We used in-house software based on MATLAB (MathWorks, Natick, MA, USA) to synchronize the motion tracking data from the sensor attached to the brush moving in mid-air with the input from the sliding bar representing the participant’s real-time illusion vividness as well as the skin conductance measurements. The subjective rating and skin conductance data were down-sampled to the frequency of the motion tracking device, namely 120 Hz. As a result, any given data point in 3-D space was assigned an illusion vividness value between -3 and +3. Finally, the location of rubber hand itself within the coordinate system was determined by systematically moving the motion sensor along each of the five fingers of rubber hand surface, the results of which are shown in Fig 3A. The onset and offsets of the approaching movements and threat events were flagged in the data file by the experimenter through the pressing of different foot pedals.

### Statistical approach

We decided to use parametric tests for all analysis, for reasons of simplicity and because the population-level data can be assumed to be normally distributed. Furthermore, the use of non-parametric tests for the comparisons in which at least on data set did not pass a Kolmogorov-Smirnoff test of normality yielded essentially the same results as our parametric approach, which is consistent with the notion that *t* statistics are reasonable robust to non-normality [19]. The alpha level was always set to 0.05. Two-tailed test were used throughout. For ANOVAs where sphericity could not be assumed, as determined by Mauchly’s sphericity test, the degrees of freedom were corrected using Greenhouse-Geisser estimates of sphericity. For our *a priori* planned comparisons, we did not adjust the alpha level to correct for multiple comparisons. For our *post hoc* tests, however, we used the Bonferroni correction for multiple comparisons. Parametric tests were analyzed using SPSS 23.0. Bayesian analyses were conducted using an online Bayes factor calculator (http://pcl.missouri.edu/bayesfactor) based on the BayesFactor package for R [20,21].

## Results

To examine the potential role of tactile expectations in magnetic touch illusion, we first analyzed the average ratings on the magnetic touch (S1-S2) and ownership questionnaire statements (S5-S6). The *visuo-tactile* condition was associated with significantly higher ratings of magnetic touch and ownership sensations compared to the *visual only—brush approaching*, *visual only—hand approaching* and *visual only*—*hand static* conditions respectively (magnetic touch statements: *t*_*23*_ = 4.40, p<0.001, *d* = 1.12; *t*_*23*_ = 3.04, p = 0.006, *d* = 0.72; *t*_*23*_ = 3.71, p = 0.001, *d* = 0.97; and ownership statements: *t*_*23*_ = 3.03, p = 0.006, *d* = 0.67; *t*_*23*_ = 2.76, p = 0.011, *d* = 0.69; *t*_*23*_ = 2.29, p = 0.031, *d* = 0.66; two-tailed paired *t*-tests; Fig 2A). These results suggest that the illusion was successfully induced in the *visuo-tactile* condition and that tactile expectations alone was not sufficient. As shown in Fig 2B and 2C, this subjective difference was mirrored in greater threat-evoked SCRs in the *visuo-tactile* compared to the *hand approaching* (mean difference±SEM: 0.039±0.015 μS; *t*_*23*_ = 2.54, p = 0.018, *d* = 0.52), *hand static* (mean difference±SEM: 0.041±0.019 μS; *t*_*23*_ = 2.13, p = 0.044, *d* = 0.44) and *visual only* conditions (mean difference±SEM: 0.029±0.016 μS; *t*_*23*_ = 1.85, p = 0.078, *d* = 0.38).

To investigate the basic tactile expectation effect previously reported [4–6], we compared the *visual only—hand approaching* and *visual only—hand static* conditions. In contrast to previous findings, we found no significant differences in terms of mean questionnaire ratings (magnetic touch statements: *t*_*23*_ = 1.45, p = 0.160, *d* = 0.30; ownership statements: *t*_*23*_ = -0.211, p = 0.834, *d* = -0.04; Fig 2A) or threat-evoked SCR (mean difference±SEM: 0.002±0.013 μS; *t*_*23*_ = 0.17, p = 0.864, *d* = 0.04) (Fig 2B and 2C). A similar result was obtained when analyzing the questionnaire statement 5, *“It felt as if the rubber hand were my hand”*, separately (*t*_*23*_ = 0.28, p = 0.780, *d* = 0.04, two-tailed paired *t*-test). In order to quantify the support for the null hypothesis, the data were also examined by estimating a Bayes factor using the Jeffreys–Zellner–Siow *t* test (using the default scale *r* = 1.0) [20,21], comparing the fit of the data under the null hypothesis and the alternative hypothesis. An estimated Bayes factor (null/alternative) suggested that the magnetic touch statement data were 2.40:1, the ownership statement data 6.24:1, and threat-evoked SCR data were 6.29:1, all in favor of the null hypothesis. Thus, there was substantial evidence in favor of the null hypothesis, meaning that the observed data were more likely to occur under a model without including an effect of tactile expectations, rather than a model with it. This failure to replicate earlier findings speaks against the notion the visually-induced tactile expectations play a role in the generation of the rubber hand and magnetic touch illusions [13].

The real-time magnetic touch ratings and continuous skin conductance recordings, which were synchronized with the motion tracking data, allowed us to examine changes in subjective magnetic touch and level of physiological stress as a function of the distance between the visual stimuli and the rubber hand (Fig 3A). Furthermore, it permitted an attempt to replicate the findings that the seen approach of the visual stimulus toward the rubber hand is coupled with a linear increase in skin conductance, reflecting tactile expectations [4,13]. Specifically, for each participant and condition, we averaged the magnetic touch rating and level of skin conductance for every 10-cm-interval between the distances 60 to 3 cm (“55 cm” represented the average across 51–60 cm, “45 cm” represented the average across 41–50 cm, and so forth; see Methods for details). For the *hand static* condition, in which no approaching motion was performed, we instead averaged the ratings and skin conductance for every 5-second interval between 0 and 30 s. At the group-level, the data was entered into two separate 4×6 repeated measures ANOVAs with the factors condition and distance (55 cm, 45 cm, 35 cm, 25 cm, 15 cm, 5cm). For the real-time subjective ratings, we observed significant effects of condition (F_1.29,29.8_ = 30.95, p<0.001, η_p_^2^ = 0.65) and distance (F_1.24,28.5_ = 27.05, p<0.001, η_p_^2^ = 0.70) as well as their interaction (F_3.47,79.7_ = 18.88, p<0.001, η_p_^2^ = 0.86). As can be seen in Fig 3B, these effects were driven by the presence of illusory magnetic touch in the *visuo-tactile* condition—starting at approximately 30 cm—and an absence of the illusion in the other three conditions, all of which featured solely visual stimulation. Thus, the subjective experience of the illusion was dependent on concurrent visual and tactile stimulation.

As for the skin conductance results, there was a significant effect of condition (F_3,69_ = 4.62, p = 0.005, η_p_^2^ = 0.167) and distance (F_2.32,53.4_ = 27.91, p<0.001, η_p_^2^ = 0.55), but no significant condition×distance interaction effect (F = _4.68,108_, p = 0.090, η_p_^2^ = 0.08). Visual inspection of the skin conductance over distance graph (Fig 3C and 3D) showed that the main effect of condition was driven by a small increase in skin conductance in the *visuo-tactile* (mean±SEM: 1.93±0.035μS) compared to the *visual only-brush approaching* (mean±SEM: 1.83±0.035μS), *visual only—hand approaching* (mean±SEM: 1.90±0.035μS) and *visual only—hand static* conditions (mean±SEM: 1.84±0.035μS). The main effect of distance was driven by a linear decrease in skin conductance over time, which did not significantly differ across conditions (Fig 3D). This result is consistent with a negative baseline drift over the time course of each trial, which is commonly observed in skin conductance research. *Post hoc* pair-wise *t*-tests among all conditions showed that the mean skin conductance level (across all distances) in the *visuo-tactile* condition was significantly higher than in *hand static* (t_23_ = 3.81, p = 0.005, *d* = 0.78); however, all other pair-wise comparisons were non-significant (p>0.05, paired two-tailed *t*-tests). Notably, there was no significant condition×distance interaction between the key tactile expectation condition (*visual only—hand approaching*) and its control (*visual only—hand static*): F_2.32,53.4_ = 0.843, p = 0.451, η_p_^2^ = 0.035; 2×6 repeated measures ANOVA), which is in contrast to the results of [4]. In summary, we failed to replicate the finding that the approach of a visual stimulus toward a rubber hand placed in an anatomically congruent condition is coupled with an increase in level of skin conductance [4]. See S1 Data for raw data.

## Discussion

We used the magnetic touch illusion and a combination of motion tracking, real-time subjective ratings and skin conductance responses to investigate the role of tactile expectations in the process attributing ownership to limbs. In summary, the results showed that the illusion is dependent on concurrent visual and tactile stimulation and that tactile expectations alone are not sufficient to induce either illusory ownership or a sense of magnetic touch, even when the trajectory of the visual stimulus is along a path compatible with a potential collision of both the real and rubber hands [13]. Furthermore, we found no evidence, neither in terms of subjective ratings nor skin conductance responses, for the notion that tactile expectations can induce ownership of an artificial limb, even when using the same key elements of the experimental setup of a previous study that reported the existence of such an effect [4]. These findings emphasize the importance of multisensory correlations for the emergence of limb ownership and contradict the hypothesis that sensory predictions of upcoming tactile events are sufficient to determine whether or not a limb belongs to the self.

In this study, we adapted the magnetic touch illusion setup using a vertical displacement of the real and rubber hands in order to maximize potential tactile expectation effects. This modification was based on the hypothesis that the trajectory of the visual stimulus needs to be along a path compatible with a potential collision of both the real and rubber hands [13]. However, one could question the theoretical grounds for this ‘common collision path’ hypothesis for two main reasons. First, in the original tactile expectation study in which a significant effect on ownership was detected [4], the participants’ real hand was placed below a table that represented a solid barrier between the (real) hand and the approaching object. Thus, even though the trajectory of the visual stimulus was along a vector pointing toward the real hand, there was not even a potential possibility for a collision with real hand, given that the object’s path does not take a detour around the edges of the table along different vectors. Second, the approaching object in [4] stopped 15 cm above the rubber hand, which was 35 cm above the real hand (the distance between the real and rubber hands was 20 cm). Thus, the approaching object never entered the theoretical limits of the real hand’s peripersonal space, which has been estimated to extend approximately 35 cm from the hand [22]; or, at best, stopped right at its edge. The conclusion that “the approaching stimulus must fall within the participant’s peripersonal space […] for tactile expectation to exert this effect [inducing ownership]” [4] is therefore questionable. If anything, the approaching visual stimulus must fall within the peripersonal space of the rubber hand—and not the real hand—for tactile expectation to induce ownership. In light of these considerations, the necessity for a ‘common collision path’ of the approaching stimulus for both the rubber hand and the real hand appears questionable.

Here, we found that tactile expectations cannot induce the magnetic touch illusion, even when a ‘common collision path’ is used. Our failure to replicate the original tactile expectation effect on ownership [4], despite using the same key elements of the experimental setup [4] and a greater number of participants (n = 24 versus n = 15), is in need of an explanation. First, it should be noted that the questionnaire results are not directly comparable across the two studies. Ferri et al (2013) used a 21-item questionnaire adapted from [23]. Neither the ratings of individual statements nor the specific statements used were reported. Instead, only the mean ratings of four clusters of statements, the most relevant being the “embodiment cluster,” are reported. The embodiment cluster comprised ten statements [4] that probe different aspects of embodiment, ranging from ownership (e.g., *“it seemed like the rubber hand was my hand”*), agency *(*e.g., *“it seemed like I was in control of the rubber hand”*) and position (e.g.,*“it seemed like my hand was in the location where my hand was”*) [23]. It is therefore unclear how high the participants actually rated the key ownership statement “*it seemed like the rubber hand was my hand*,*”* which was used in the present study. Nevertheless, given that the average rating of the embodiment cluster statements was 2.1, it is likely that the participants rated the key ownership statement higher than the participants in our study did (0.25; Fig 2A). Furthermore, no control statements were used [4], which makes it difficult to rule out the well-known effect of participants trying to comply with the task by giving high ratings in conditions in which they believe that the experimenter ‘wants’ them rate highly [24]. Finally, all participants in our study were exposed the original rubber hand illusion [15], providing them with an “internal reference” of what illusory limb ownership feels like, which might generate more accurate estimations of illusion strength in the tactile expectation conditions.

The findings of the present study are also incompatible with those of a recently published study, which reported that rubber hand ownership was induced by tactile expectations when a rubber hand was vertically, but not horizontally, displaced with respect to the real hand [25]. Specifically, Experiment 2 in [25] tested 15 participants and found that performing 60 short, consecutive approaching movements toward a rubber hand, which was displaced vertically relative the real hand, was associated with an illusion of ownership (mean rating of embodiment cluster [23] statements = 1.5). However, this effect on subjective ratings was not supported by any behavioral or physiological proxy of body ownership, such as proprioceptive drift data or threat-evoked SCR, and should therefore be interpreted with caution. Furthermore, the key contrast *horizontal* versus *vertical* rubber hand displacement was, in fact, not statistically significant (*t*_*14*_ = -2.58, p = 0.130). Because of the lack of a ‘visual only’ control condition in which vision and proprioception were matched but no approaching movement was performed, it is impossible to rule out that the effect observed in the vertical ‘tactile expectation’ condition was due to a visuo-proprioceptive match *per se*. Further studies are needed to clarify the minimal conditions required to induce the rubber hand illusion without correlated visual and tactile stimulation, and whether there is something special about visual movement approaching the rubber hand.

Bayesian analysis of the threat-evoked SCR as well as the questionnaire data for the key tactile expectation contrast (*visual only—hand approaching* versus *visual only—hand static*) showed substantial evidence in favor or the null hypothesis. Moreover, we failed to replicate the finding that tactile expectations is associated with an increase in skin conductance as the approaching visual stimulus enters the peripersonal space of the rubber hand [4]. In our experiment, the skin conductance decreased slowly and linearly with distance (or time) in all conditions, including the key tactile expectation condition *visual only—hand approaching* (see Fig 3C). The changes in SCR between 55 cm and 5cm ranged from -3.3% to -2.2% in all conditions (Fig 3D). In stark contrast, Ferri *et al* (2013) reported a dramatic change in skin conductance as a function of distance in their corresponding *visual only—hand approaching* illusion condition. Specifically, this previous study found that the average skin conductance was 0.41μS at 60cm and 0.88μS at 15cm (i.e., a 115% increase) in the illusion condition, which should be compared to the corresponding skin conductance change in the three control conditions which ranged from -7% to +23% (Table 1 and Fig 3 in [4]). It should be noted that a bandpass filter (0.01–0.5 Hz) was applied to the data in [4], which removes slow baseline drifts like the one observed in the present study. Furthermore, the skin conductance value for each of the four distances included in [4] (60 cm, 45 cm, 30 cm and 15 cm) was defined as the maximum value of the first principal component of the skin conductance data within a 10 s time-interval after the experimenter’s hand passed by optical sensors positioned at each distance. Because the experimenter’s hand moved at the speed of 2 cm/s, the value for each distance could potentially reflect skin conductance changes occurring up to 20 cm below each distance. Despite these differences in analysis approaches, the absence of an interaction between condition and distance in our data is incompatible with the findings in [4].

In [4], the change in skin conductance in the illusion condition was found to correlate significantly with the mean embodiment ratings [4]. However, these previous skin conductance results are difficult to interpret conceptually, because the SCR was not threat-evoked [4]. Indeed, the established use of SCR to measure illusory body ownership is by quantifying the SCR evoked by a physical threat directed toward the artificial body part [17,18,26–30]. The rationale is that participants who truly experience the rubber hand as part of the self, their autonomic nervous system reacts as if it were their real body being threatened [17,26]. However, the rationale behind why ownership of an artificial limb *per se* should lead to autonomic arousal is unclear. In light of this in conjunction with the failure to replicate the main SCR results of [4] in the present study, we propose that the hypothesized tactile expectation effect on skin conductance should be considered preliminary at best, until further positive replications or new evidence is presented.

It could be the case that the exposure the original rubber hand illusion or the inclusion of a visuo-tactile condition in the current study somehow affected the participants’ expectations in the conditions that involved only visual stimulation, which could potentially explain the our failure to replicate previous findings [4]. From the present data, this alternative interpretation cannot be ruled out. However, the tactile stimulation employed here consisted of strokes or ‘taps’ with a paintbrush and was always coupled with the visual input of a paintbrush. It appears unlikely that this type of visuo-tactile stimulation should substantially change the expectation associated with a distinct visual stimulus (viewing the experimenter’s hand moving toward the rubber hand). Future investigations of the effect of tactile expectations on ownership should examine this issue. It would also be a great value to quantify participants’ explicit expectations (e.g., *“Did you expect that the viewed motion would results in a touch on your hand*?*”*), which neither the present study nor previous studies [4–6] have done.

In contrast to the negative results in our unisensory conditions, the multisensory *visuo-tactile* condition was associated with the experience of both illusory magnetic touch and rubber hand ownership. These effects were supported by higher subjective ratings as well as increased threat-evoked SCR in the *visuo-tactile* compared to the three ‘visual only’ control conditions. In accordance with previous findings [12,14], we found that the magnetic touch illusion was dependent on concurrent visual and tactile stimulation and sets in approximately at the theoretical boundary of peripersonal space [22]. These results reinforce the notions that multisensory integration within peripersonal space is a key mechanism for the emergence of body ownership [7–11] and that correlated sensory stimulation from at least two modalities is necessary to induce the rubber hand illusion [15,18,27,31].

The present study extend the conflicting results of previous studies [4–6,12,13] and contribute to a better understanding the role of tactile expectations in the emergence of body ownership. In light of the absence of a robust tactile expectation effect in the present data, we propose that tactile expectations alone should not be considered sufficient to induce a sense of ownership over artificial limbs, until further, more convincing evidence is presented.

## Supporting information

S1 Data Raw data. Questionnaire, threat-evoked SCR, and continuous skin conductance and subjective ratings data.
(XLSX)

S1 Fig Questionnaire results for the original rubber hand and magnetic touch illusions. See Table 1 for statements. The error bars denote the SEM.
(PDF)

S2 Fig Motion tracking results for the knife threat event. The sensor was attached to the experimenter’s hand that held the knife and made a ‘cutting’ motion by sliding the knife over the rubber hand. The rubber hand’s surface is indicated with black-colored data points. In the right panel, a series of five sequential images illustrating the entire threat event has been overlaid the motion tracking data for display purposes. The motion took approximately 2 s. The data shown is pooled from all participants, and each individual trial is assigned a unique (random) color. As revealed by visual inspection of this data, the knife motion was performed very consistently by the experimenter from trial to trial.
(TIF)
