# Supplementary figures and images for: Multisensory correlations—Not tactile expectations—Determine the sense of body ownership
Source: PLoS One. 2019 Feb 28;14(2):e0213265. doi: 10.1371/journal.pone.0213265 (PMC6394992; doi:10.1371/journal.pone.0213265)

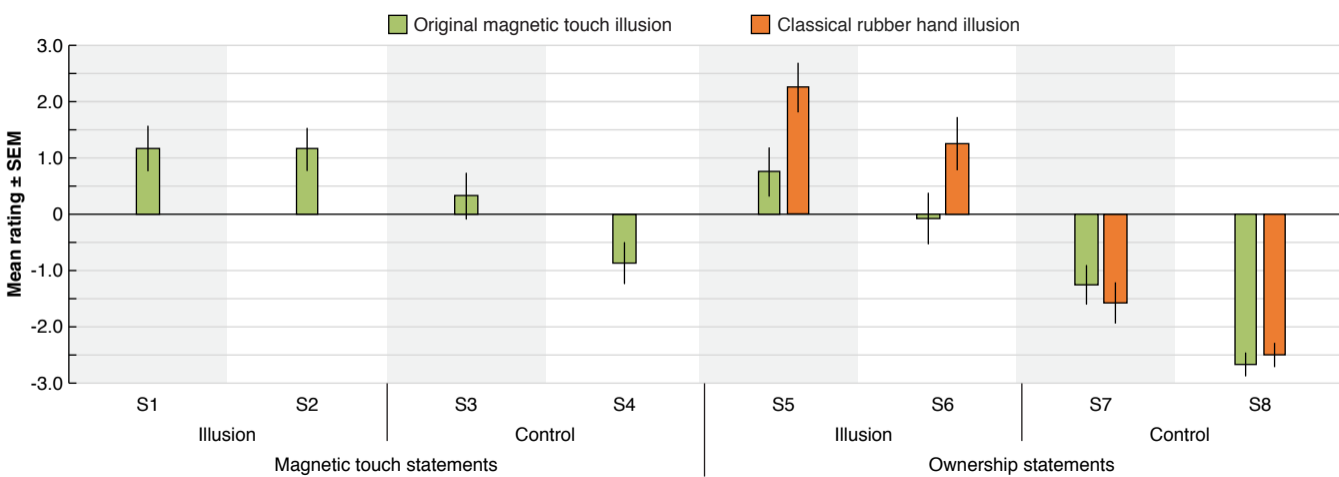

Supplement: S1 Fig — See Table 1 for statements. The error bars denote the SEM. (PDF) [file pone.0213265.s002.pdf]

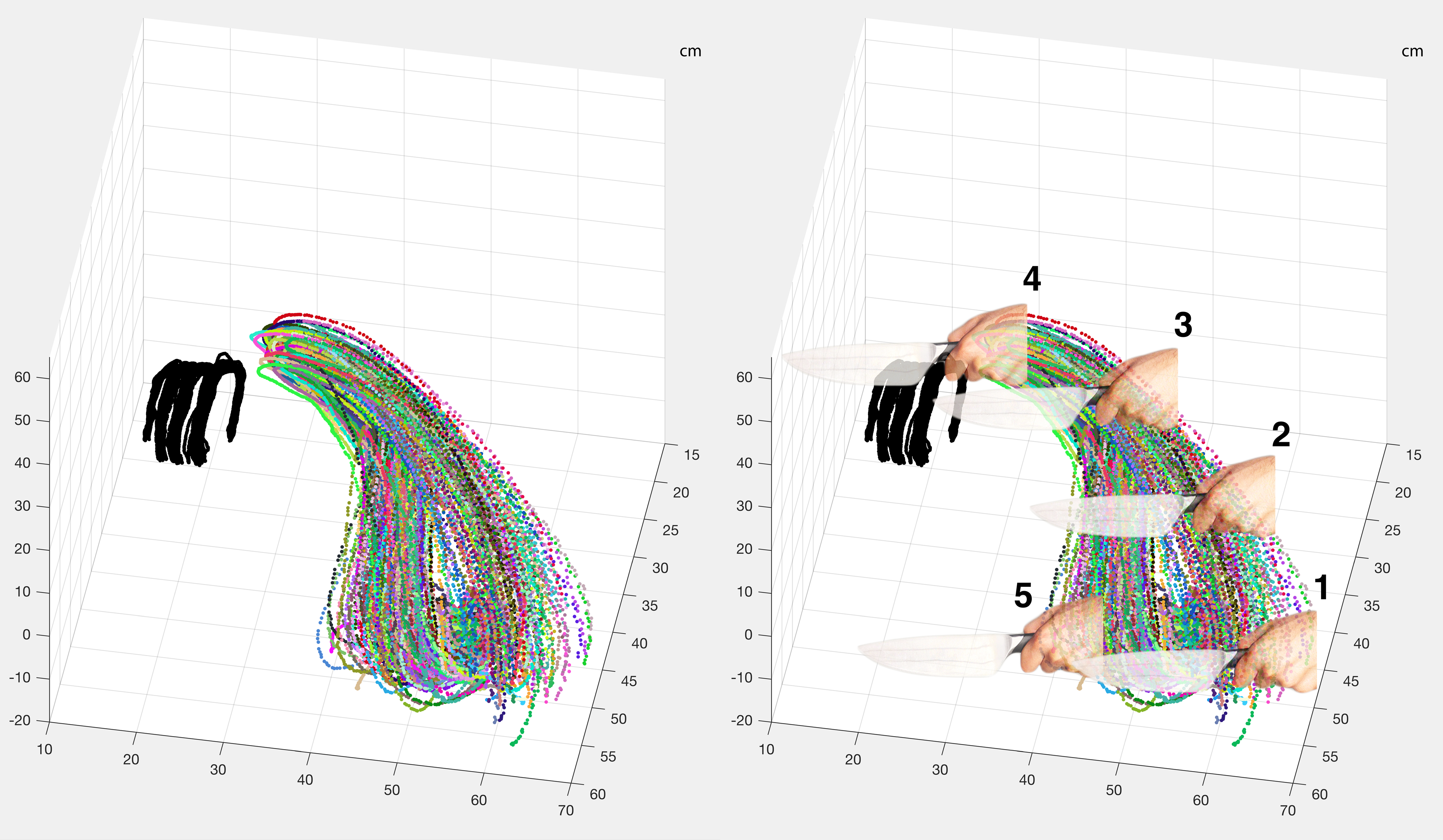

Supplement: S2 Fig — The sensor was attached to the experimenter’s hand that held the knife and made a ‘cutting’ motion by sliding the knife over the rubber hand. The rubber hand’s surface is indicated with black-colored data points. In the right panel, a series of five sequential images illustrating the entire threat event has been overlaid the motion tracking data for display purposes. The motion took approximately 2 s. The data shown is pooled from all participants, and each individual trial is assigned a unique (random) color. As revealed by visual inspection of this data, the knife motion was performed very consistently by the experimenter from trial to trial. (TIF) [file pone.0213265.s003.tif]
